# Monolithically integrated broad-band Mach-Zehnder interferometers for highly sensitive label-free detection of biomolecules through dual polarization optics

**DOI:** 10.1038/srep17600

**Published:** 2015-12-02

**Authors:** A. Psarouli, A. Salapatas, A. Botsialas, P. S. Petrou, I. Raptis, E. Makarona, G. Jobst, K. Tukkiniemi, M. Sopanen, R. Stoffer, S. E. Kakabakos, K. Misiakos

**Affiliations:** 1 Immunoassay/Immunosensors Lab, Institute of Nuclear & Radiological Sciences & Technology, Energy & Safety, NCSR “Demokritos”, 15310 Athens, Greece; 2 Optical Biosensors Lab, Institute of Nanoscience and Nanotechnology, NCSR “Demokritos”, 15310 Athens, Greece; 3 Jobst Technologies GmbH, 79108 Freiburg, Germany; 4 VTT, Espoo, FI-02044 VTT, Finland; 5 PhoeniX BV, 7521 PA Enschede, The Netherlands

## Abstract

Protein detection and characterization based on Broad-band Mach-Zehnder Interferometry is analytically outlined and demonstrated through a monolithic silicon microphotonic transducer. Arrays of silicon light emitting diodes and monomodal silicon nitride waveguides forming Mach-Zehnder interferometers were integrated on a silicon chip. Broad-band light enters the interferometers and exits sinusoidally modulated with two distinct spectral frequencies characteristic of the two polarizations. Deconvolution in the Fourier transform domain makes possible the separation of the two polarizations and the simultaneous monitoring of the TE and the TM signals. The dual polarization analysis over a broad spectral band makes possible the refractive index calculation of the binding adlayers as well as the distinction of effective medium changes into cover medium or adlayer ones. At the same time, multi-analyte detection at concentrations in the pM range is demonstrated.

Miniaturized bioanalytical devices find applications in areas vital to public interest, such as personalized health care^1^ and detection of pathogens in food^2^. Optical detection is advantageous over other sensing methods as the optical frequency regime of operation and the galvanic isolation of the transducer from the excitation and detection electronics suppress unwanted parasitic currents and signal drifts. Numerous optical detection principles, such as surface plasmon resonance (SPR)^3^, optical waveguides^4^,^5^, grating couplers^6^, Mach-Zehnder^7^,^8^,^9^ and Young interferometers^10^, microring resonators^11^,^12^, resonant mirrors^13^, and reflectometric interference spectroscopy sensors^14^,^15^ have been explored. A comparison of the various optical configurations favours waveguide based integrated interferometers as the photons there probe the biomolecular adlayer hundreds or thousands of times compared to a couple of times in reflectometric interference spectroscopy or ellipsometric methods. As a result, interferometric sensors based on planar waveguides are far more sensitive compared to those based on free-space optics^16^,^17^.

The sensitivity enhancement and immunity to parasitics make planar waveguide-based interferometric devices ideal for label free testing. However, the inability to monolithically integrate on the same chip active optical components along with the interferometric sensing elements has so far hindered the proliferation of such devices. Very recently^18^,^19^, interferometric silicon chips were presented with monolithically integrated light emitting devices (LEDs), as shown in Fig. 1a. The LEDs employed are silicon avalanche diodes that emit white light when biased beyond their breakdown voltage^20^. The LEDs are coupled to co-integrated monomodal waveguides that are shaped as Mach-Zehnder interferometers (MZIs) through mainstream silicon technology. The silicon LEDs feed their white light to the MZIs, and following the second Y- junction of the MZI, the modulated output spectrum is monitored by an external spectrometer (Fig. 1a). This Broad-Band Mach-Zehnder Interferometry (BB-MZI) employs broad-band light and greatly enhances the spectral shifts^18^ compared to other type of single-wavelength interferometers, like ring resonators. In a ring resonator, the spectral shifts upon effective medium changes are inversely proportional to the wavelength derivative of the *N*_*s*_*/λ* ratio, where *N*_*s*_ is the resonator effective refractive index. In a MZI, with reference and sensing arm effective refractive indices equal to *N*_*r*_ and *N*_*s*_, respectively, the spectral shifts are inversely proportional to the wavelength derivative of the (*N*_*r*_*-N*_*s*_)/*λ* ratio^18^. The latter is about two orders of magnitude smaller than the former one, resulting in equally higher spectral shift sensitivities. Such a spectral shift enhancement in association with the broad-band nature of the spectral output makes possible the use of portable spectrometers as high resolution detectors enabling thus high performance Point-of-Need (PoN) determinations^21^,^22^,^23^. By recording the entire spectrum emitted by the chip, wavelength interrogation is possible over a broad spectral range with substantial benefits in simultaneous dual polarization analysis and, also, in the limit of detection. Such attributes have already been demonstrated in the case of a single interferometer operating as a cover medium refractive index sensor^18^. Here, we extend the work to multiplexed protein detection and bound protein refractive index determination on chips carrying arrays of MZIs, as illustrated in Fig. 1b. The refractive index determination is based on the simultaneous TE and TM monitoring and the broad-band nature of the waveguided light. The recorded spectra are analyzed both in the Fourier Transform domain and in the deconvoluted direct wavelength domain. The former allows sensitive protein detection (in the pM range), while the latter provides wavelength resolved dual polarization information that reveals the protein’s layer refractive index^24^. The bioanalytical and multi-analyte detection capabilities of MZI array silicon chips are exemplified through spotting of the sensing arms with mouse gamma globulins, biotinylated bovine serum albumin, and bovine serum albumin (to serve as blank) and monitoring the responses upon reaction with anti-mouse IgG antibody and streptavidin, respectively. For the supply of the reagents an appropriate fluidic cell was designed and attached on the chip, Fig. 1c, at wafer level scale which greatly enhances the fabrication of the complete biochips. After silicon wafer dicing the biochips were encapsulated on a cartridge and inserted in the docking station. The measuring apparatus include the power and control electronics, the off-chip fluidic circuit, the docking station, a dedicated optical module and a miniaturized spectrometer. The control electronics provided for the integrated LED multiplexing so that the 10 interferometers could be interrogated sequentially with a delay of a few ms in between. This way multi-analyte determinations are possible.

## Simultaneous dual polarization detection: principle of operation

The broad-band nature of the waveguided light allows the compression of sufficient information in a single spectrum so that deconvolution of both polarizations without employing polarizers and separate recordings is possible. At the same time, the monitoring of spectral shifts over a broad spectral range provides clues on whether the shifts are caused by cover medium or adlayer changes. To see how polarization and effective medium deconvolution is possible, let us consider the transfer function *T(λ)* of a MZI:


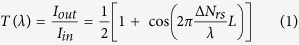


where the term Δ*Ν*_*rs*_ is the difference between the reference and sensing arm effective refractive indices, Δ*Ν*_*rs*_ = *N*_*r*_ *−* *N*_*s*_, and *L* is the exposed length of the sensing arm. The terms *I*_*in*_ and *I*_*out*_ are the input and output optical powers, respectively. The striking simplicity of the above equation is to be contrasted with complex expressions regarding the broad-band response of ring resonators. In the cosine argument, Δ*N*_*rs*_*/λ* is the key parameter of the MZI. The effective index difference Δ*Ν*_*rs*_ provides for a higher flexibility in tailoring the spectral response compared to the single term effective index of a standard ring resonator. Through appropriate engineering of the sensing arm with respect to the reference arm the Δ*N*_*rs*_*/λ* term can become almost linear over a wide spectral range^18^. This is achieved by etching away a certain thickness (a few nm) of the exposed sensing arm silicon nitride core through a reactive ion etching process step. Here, the sensing arm core thickness was thinned down to 150 nm compared to the reference arm core thickness of 167 nm.

Under conditions of linearity:


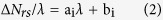


while


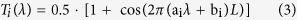


where i = 1 stands for TE and i = 2 stands for TM. The slope factor a_i_ determines the interference fringes frequency, while the intercept b_i_ determines the position of the sinusoid along the wavelength axis.

If a broad-band spectrum is fed at the input, the spectral shift analysis of sinusoidal outputs can be efficiently handled by Fourier Transform (FT) with the added benefits of reduced noise and signal fluctuation immunity^18^. The two polarization characteristic sinusoids modulate the recorded spectra and the interference fringes with two distinct frequencies which appear in the 600–900 nm spectral range as shown in Fig. 2a: a smaller frequency corresponding to TE and a higher one with lower amplitude corresponding to the TM polarization. In Fig. 2 the cover medium makes the transition from water to a water-isopropanol mixture (16.66% v/v isopropanol) and then back to water. The Fourier transform of the transmission spectrum, as shown in Fig. 2b, has two discrete bands each containing a sharp peak: the lower frequency corresponds to TE and the higher to TM. The positions of the peaks in the Fourier transform domain are 2πa_i_*L*, while at the peaks the phase is 2πb_i_*L.* A change in Δ*Ν*_*rs*_ due to a cover medium transition, n_c_, and/or an adlayer growth will result solely from the change Δ*Ν*_*s*_ of the sensing arm effective refractive index:


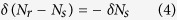


Considering first a cover medium transition, an increase in n_c_ keeps the slope factor a_i_ practically constant as the induced *δΝ*_*s*_*/λ* ratio is nearly wavelength independent^18^. Simultaneously, the intercept b_i_ drops by δb_i_ due to a decreasing *N*_*r*_*-N*_*s*_ difference, and the entire spectrum moves to the blue by 2πδb_i_*L*. The phase plots of either polarization for a cover medium transition of Δn_c_ = 1.2 × 10^−2^ RIU is shown Fig. 2c,d. The two polarizations are monitored simultaneously by keeping track of the phases at the TE and TM peaks in the Fourier transform domain. A 2π shift for the TE or TM polarization means the interference spectrum moves by one full period.

The entire spectra for either polarization can be deconvoluted and monitored separately. This is done by converting through inverse FT the TE and, separately, the TM regions in the FT domain back to the wavelength domain. This way, at each spectrometer channel the deconvoluted TE and TM spectral values are assigned and monitored over time. Consequently, the simultaneous monitoring of a large number of independent single wavelength MZIs over a wide spectral range and for either polarization is possible as shown in Fig. 3. For a channel centered at wavelength λ the deconvoluted TE and TM signals will complete a full sinusoidal swing if the sensing arm effective refractive index changes by *δΝ*_*si*_* = λ/L*, as Eq.(1) indicates. Alternatively, a *δΝ*_*si*_ effective index change will induce a swing δφ in rads given by


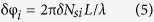


where the subscript i = 1, 2 stands for TE, TM, respectively. Single wavelength swings as a function of the cover medium transitions of Fig. 2 are shown in Fig. 3a,b. From the deconvoluted TE and TM transitions, the measured spectrum of δφ_i_ can be plotted and compared to theoretical calculations of the right hand side in eq. (5), 2π*δΝ*_*si*_ *L/λ*, as shown in Fig. 3c. The calculations of the TE and TM mode effective indices were obtained from the FemSIM software package (SYNOPSYS). The excellent agreement of the measured and calculated values, point to the accuracy of the mode deconvolution algorithm and the ability to simultaneously measure the TE and TM responses. In terms of signal-to-noise ratio, the Fourier Transform phase monitoring (Fig. 2c,d) is preferential as wavelengths are embedded in the phase information. However, the deconvoluted single wavelength MZIs (Fig. 3) provide additional information on the TE vs TM phase shifts that are important in the optical characterization of the adlayers, as will be pointed out later in this paper.

## Protein detection: Sensitivity, detection limits and model assays

An adlayer of proteins on the sensing arm introduces a *δΝ*_*s*_*/λ* function that has a different wavelength dependence compared to the same function under cover medium changes displayed in Fig. 3c. The calculated wavelength dependence of *δΝ*_*s*_*/λ* for a typical example of a 5 nm-thick protein adlayer with a refractive index of 1.46 is shown in Fig. 4. In the TE case, the adlayer induced *δΝ*_*s*_*/λ* ratio drops with λ, as opposed to an increasing ratio in the cover medium case (Fig. 3c). In the TM case, the *δΝ*_*s*_*/λ* drop with λ is much faster in the adlayer case than in the cover medium one. This is due to the fact that the longer the wave function, the more it penetrates into the cover medium with an adlayer of a few nm in thickness. Concluding on Figs 3c and 4, the broad band response, especially of TE, reveals whether a spectral shift was caused by a cover medium or an adlayer transition.

To quantitatively account for the adlayer induced spectral shifts, the corresponding *δΝ*_*s*_*/*λ ratio can be assumed nearly linear with λ, as Fig. 4 shows, and we obtain:


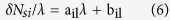


where the subscripts i and l stand for the polarization and the adlayer, respectively. From (6) and with the help of (1) and (2), the transfer function (3) in the presence of an adlayer can be rewritten as:


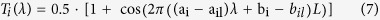


It turns out that for a protein layer of the thickness of the order of a few nm, the change in the slope factor a_i_ is rather minor and the main effect is in the phase factor b_i_. Nevertheless, as an elementary analysis of the Fourier Transform of the transfer function (7) shows, the adlayer induced phase change calculated at the fixed Fourier Transform frequency, *2π*a_i_*L* (the peak frequency in the absence of the adlayer) is


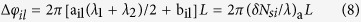


where λ_1_and λ_2_ are the lower and the upper limits of the spectral range considered and (δΝ_si_/λ)_a_, the average value, within the spectral range, of the *δN*_s_/λ ratio in (6).

The fundamental detection limit based on shot noise considerations is inversely proportional to the exposed sensing arm length (*L)*^18^:


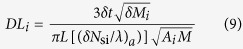


In Eq. (9), A_i_ is the input photon number per spectrometer channel and M is the number of channels in the LED spectral range. The product *A*_*i*_*M* is the total number of photons entering the MZI in each recording. In Eq. (9) is the biomolecular adlayer thickness which causes the *δN*_*s*_*/λ* ratio and *δM*_*i*_ are the number of points where the peak resonance is spread as the FWHM in the FT domain.

From Fig. 4, *(δN*_*s1*_*/λ*)_a_ = 1.8 × 10^−6^nm^−1^ (TE), *(δN*_*s2*_*/λ*)_a_ = 2.66 × 10^−6^nm^−1^ (TM) for *δt* = 5 nm and a protein layer refractive index of 1.46. Additionally, from Fig. 2a,b, for the *A*_*1*_*M* product (TE) we can take as equivalents A_1_ = 4000 and M = 200 nm/0.44 nm = 454, where 0.44 nm is the spectral width of a spectrometer channel. Also *δM*_*1*_ = 1. For TM, A_2_ = 1500, M = 454, *δM*_*2*_ = 7. Therefore, by keeping the sensing arm length *L* as a parameter, the detection limits (DL), in terms of adlayer thickness, for the two polarizations are:


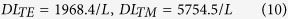


where the detection limits and L are expressed in nm. The lower detection limit for TE despite the worse performance in terms of the *δN*_*s*_* /λ* ratio compared to TM is a result of the higher signal (A) and sharper resonance peak (*δΜ*) in the Fourier transform domain. For the 600 μm long sensing arm the detection limits are *DL*_*TE*_ = 3.28 pm and *DL*_*TM*_ = 9.59 pm of adlayer, respectively. It should be noted, that longer sensing arms would further improve the detection limits. In fact, a sensing arm length of 3000 μm would result in a practical detection limit for TE and TM of about 1 pm and 2.5 pm of adlayer, respectively^18^. Considering an adlayer density of nearly 1 gr/cm^3^, the above limits translate in a surface density of 100 pgr/cm^2^ and 250 pgr/cm^2^ for TE and TM, respectively. In practical terms, however, the ultimate limit of detection limits is determined by non-specific interactions and temperature fluctuations^18^,^25^.

The protein detection limits of the proposed biosensor were investigated through the implementation of two model binding assays, namely biotinylated bovine serum albumin (biotinylated-BSA) and mouse IgG. Prior to the deposition of the biomolecules the chips were cleaned and hydrophilized by oxygen plasma treatment in order to avoid damage of the chip’s aluminum contact pads^26^ and then modified with (3-aminopropyl)triethoxysilane (APTES). In Fig. 5, the TE responses obtained from two interferometers of a chip upon probing with a 5 pM streptavidin solution are provided along with the responses of two others of the same chip coated with BSA to serve as blanks. The blank waveguides did not provide any measurable response throughout the experiments, whereas the b-BSA activated waveguides provided responses clearly distinguished from the blanks. Taking into account the signal variation of the blanks a limit of detection below 5 pM can be claimed as the concentration corresponding to 3SD of blank sensor response.

The analytical performance of the proposed interferometric chips was also investigated in the case of another model binding assay, i.e., the reaction between mouse IgG and anti-mouse IgG antibody. The calibration curves obtained for anti-mouse IgG antibody concentrations ranging from 0.025 to 66.7 nM taking into account the phase shifts in TE and TM mode are depicted in Fig. 6. The detection limits of the assay calculated as the concentration corresponding to +3SD of the blank sensors values (sensors coated with BSA) is 32 pM when the TE phase shifts are used for the preparation of calibration curve and 40 pM when the TM phase shifts are taken into account. This was due to higher variation of TM phase shifts obtained for the blank sensors as compared to respective TE phase shifts variations. The linear response of both curves extended up to 3.3 nM. The coefficient of variation (CV) of the values obtained from the different interferometers of the same chip was less than 10%, whereas the CVs of the responses obtained from different chips run in the same day or in different days did not exceed 12 and 15%, respectively.

## Protein adlayer refractive index determination

**Figure 1:**
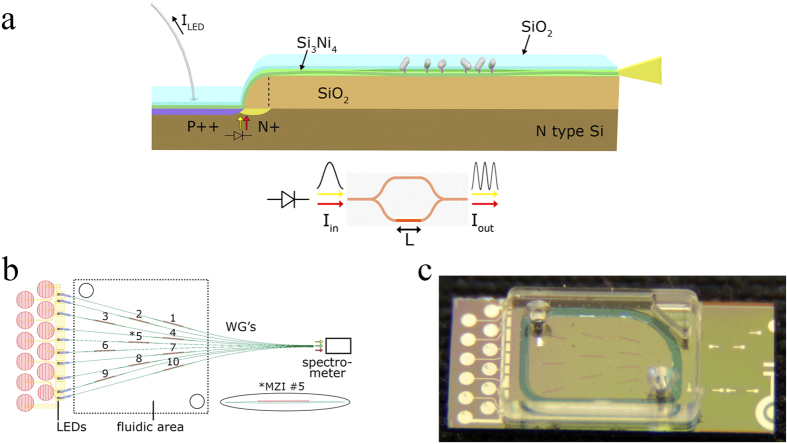
Monolithically integrated broad-band Mach-Zehnder interferometer chip. (**a**) Schematic of the biochip showing the monolithic integration of the avalanche-type LED, the Mach-Zehnder interferometer, and the silicon nitride rib waveguide. The SiO_2_ quadrant spacer with the broken line is employed to reduced waveguide bending losses. The single mode rib waveguides have a width of 1.25 μm and an etch depth of 4 nm. The sensing arm thickness is 150 nm. The chip emitting edge is coupled to an Ocean Optics, Maya 2000 portable spectrometer, through an external fiber. The inset below the main Figure illustrates the photonic diagram and interference effects on the output light. (**b**) Layout of the 10 MZIs showing the MZI routes as well as the LED positions and metal. (**c**) Chip carrying the fluidic module with the inlet and outlet holes placed diagonally for the supply of the reagents. The 10 exposed MZI arms are visible under the cover.

**Figure 2:**
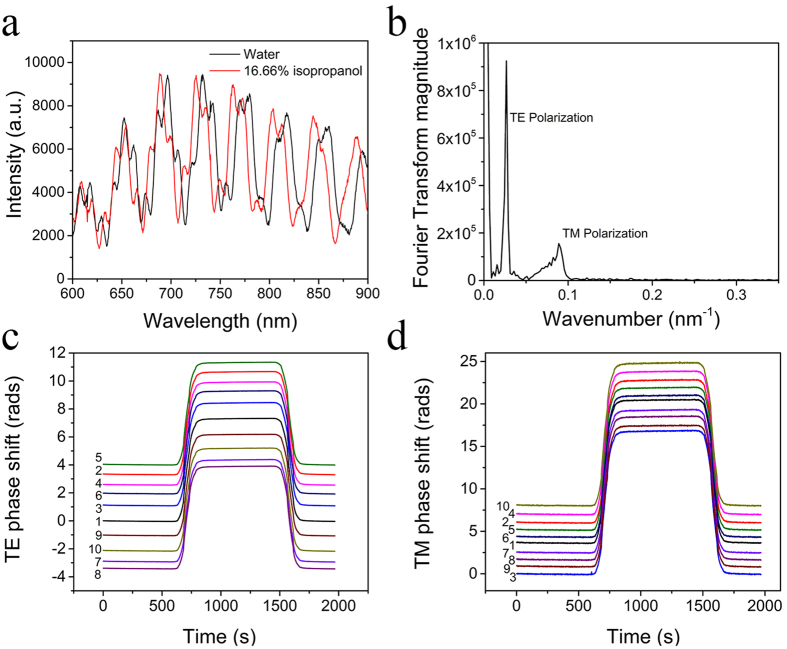
Transmission spectra and processed results. (**a**) Spectrum of the modulated output and the effect of the cover medium refractive index change. Two oscillation frequencies are obvious, a lower and a higher for the TE and TM polarization, respectively. The black curve is obtained with water as cover medium, while the red one when the cover medium is water containing 16.66% v/v isopropanol (The cover medium refractive index change is Δn_c_ = 1.2 × 10^−2^ RIU). The peaks are blue-shifted by more than a period. Here the spectrometer integration time is set at 1 s. (**b**) Fourier transform of the spectrum showing one main peak for the TE polarization and another one at a higher frequency for TM. (**c**) TE response to cover medium transition from water to 16.66% v/v isopropanol solution for all ten sensors on the chip. The isopropanol solution enters at 600 s and the transition back to water is at 1500 s. The phase change 2πδb_1_L across the MZI array is in the 7.29–7.36 rad range (±0.6% variation). (**d**) TM response for all ten sensors of the same chip to cover medium transition from water to 16.66% v/v isopropanol solution. The phase change 2πδb_2_L across the MZI array is in the 16.7–16.9 rad range (±0.6% variation). In (**c**) and (**d**) the spectrometer integration time is 0.1 s.

Chips with arrays of BB-MZIs were selectively functionalized with mouse IgG, biotinylated-BSA or BSA to explore the potential of simultaneous protein detection and refractive index analysis. Considering the chip schematic in Fig. 1b, mouse IgG, biotinylated-BSA and BSA (serving as blanks-controls) were spotted on WGs 1, 4, 7, and 10, WGs 2, 5, and 8, and WGs 3, 6, and 9, respectively. The assay results in Fig. 7 are the phase shifts of the two polarization peaks as in Fig. 2b. As expected, the response corresponding to streptavidin binding to immobilized biotinylated-BSA is much faster than that corresponding one of anti-mouse IgG antibody binding to immobilized mouse IgG because of the higher binding affinity of streptavidin to biotin. As shown, when the streptavidin solution is passed over the waveguides the mouse IgG spotted interferometers have a slight response. This is result of the non-specific streptavidin-mouse IgG interactions. This phenomenon would be supressed if molecules with very low non-specific interactions have been selected for biofunctionalization of different BB-MZIs of the same chip. The blank waveguides remained unresponsive. After 20 minutes of reaction, the TE (TM) phase shifts due to the streptavidin binding were 4.25 (5.73), 4.56 (6.87), and 4.48 (6.02) radians for interferometers 8, 5 and 2, respectively. The reaction with 10 nM anti-mouse IgG leads to smaller TE (TM) shifts for the same reaction time: 1.15 (1.74), 1.16 (1.67), 1.11 (1.79), and 1.04 (1.66) radians for interferometers 10, 7, 4, and 1, respectively. The deviation of the signal with interferometer can be attributed to the different flow rates on top of the chip. The waveguides along the inlet-outlet diagonal (Fig. 1b) get to obtain more analyte supplies compared to the others.

**Figure 3:**
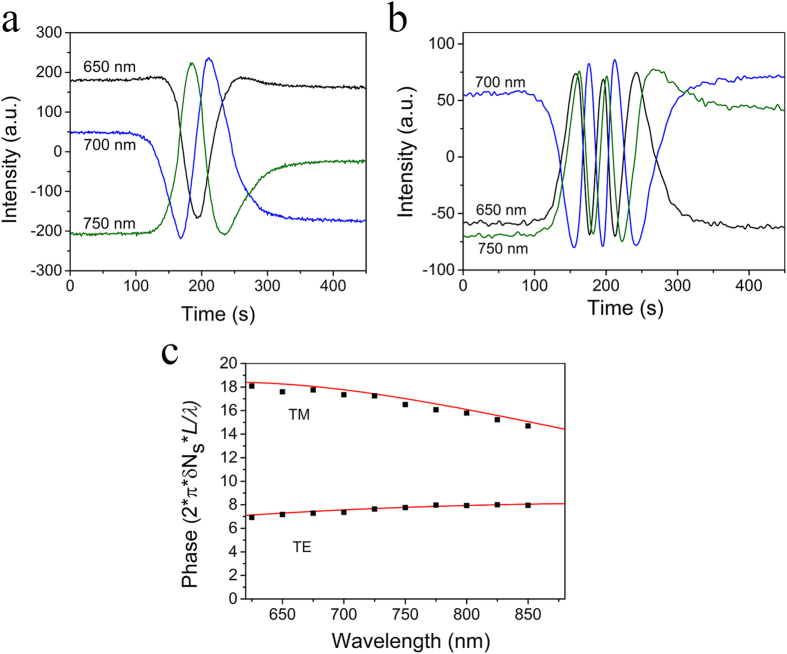
Deconvoluted spectra of TE and TM transitions with the cover medium change of Fig. 2. (**a**) Single-wavelength TE oscillators for MZI#1 at three wavelengths (650, 700, and 750 nm). (**b**) Single-wavelength TM oscillators for MZI#1 at three wavelengths (650, 700, and 750 nm). In (**a**,**b**) the DC component of the signals have been subtracted while the spectrometer integration time is set at 0.1 s. (**c**) Phase change of the deconvoluted TE and TM oscillations as a function of the wavelength (squares). The red lines are the theoretical expressions φ_i_ = 2π*δΝ*_*si*_*L/λ* with δΝ_si_ obtained from optical simulations.

**Figure 4:**
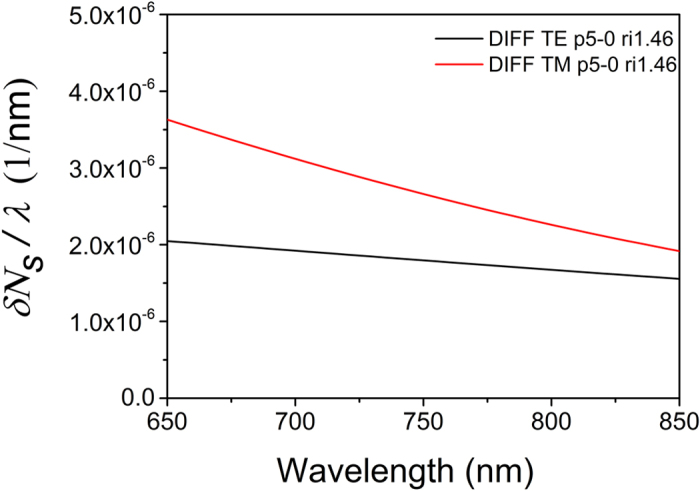
The spectrum of the *δΝ_s_/λ* ratio for the two polarizations and a 5 nm protein adlayer with a refractive index of 1.46. The thickness of the sensing arm was 150 nm, while the value of 1.34 RIU was used for the cover medium index of refraction

**Figure 5:**
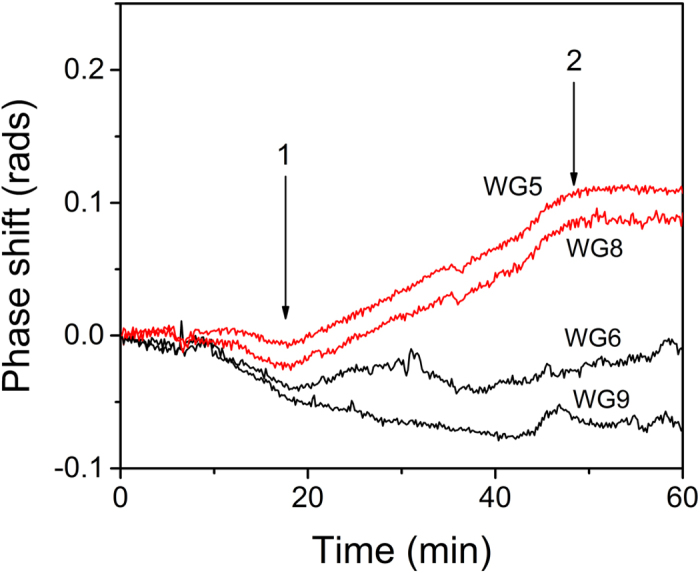
Real-time TE responses obtained from a chip spotted with biotinylated-BSA. Waveguides 5 and 8 (Fig. 1b) were spotted with biotinylated-BSA while 6 and 9 with BSA. The sequence of the solutions running over the chip surface is as follows: start to arrow 1: assay buffer; arrow 1 to 2: 5 pM streptavidin solution in assay buffer; arrow 2 to end of run: assay buffer. The flow rate was 20 μl/min.

**Figure 6:**
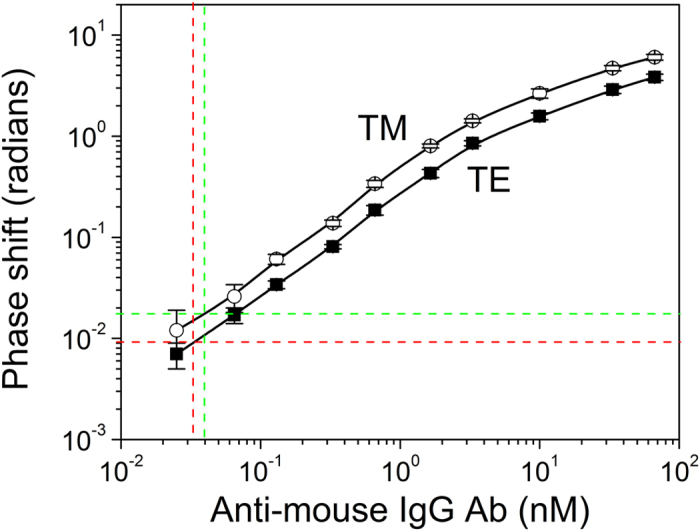
Typical calibration curves for anti-mouse IgG antibody obtained from chips spotted with mouse IgG based on phase shifts on TE (■) or TM (○) mode. Horizontal lines correspond to signals equal to 3SD of blank sensors responses and vertical lines show the corresponding detection limit based on TE (red lines) or TM (green) phase shifts. The reaction time is 20 min. Each point is the mean value of 4 chips ± SD.

**Figure 7:**
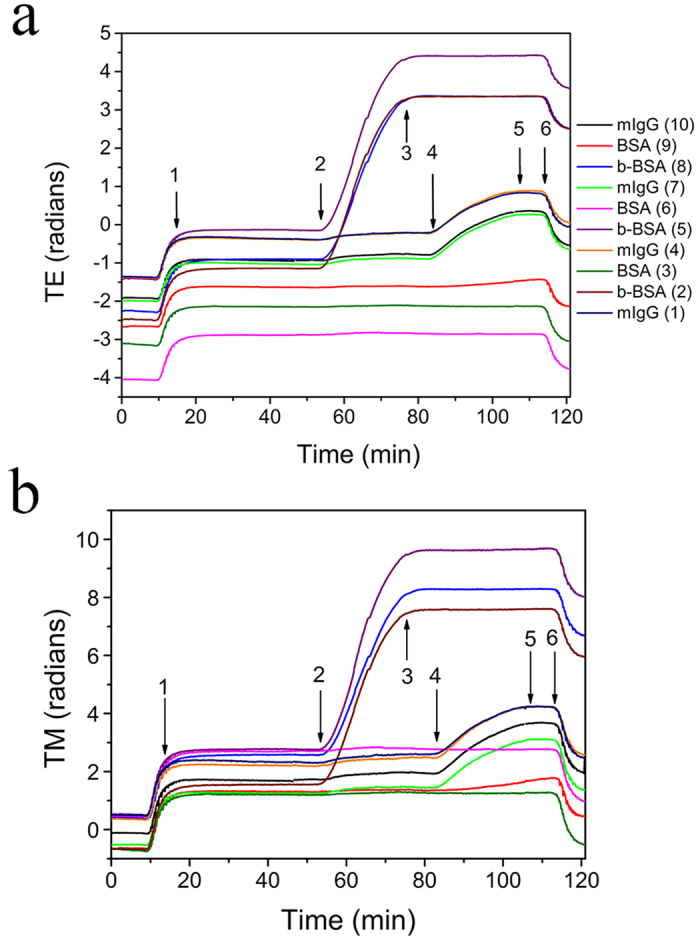
Multiplexed dual polarization response of a two analyte assay. The 10 sensing arms were split into three groups: Four out of 10 (WGs 1, 4, 7, and 10) were spotted with mouse IgG, 3 (WGs, 2, 5, and 8) with biotinylated-BSA and the rest 3 with BSA (WGs 3, 6, and 2). The plastic cover with the fluidic seal and the conical holes of Fig. 1c was placed on top of the chip and clear of the sensing arms. The encapsulated chip was inserted in a docking station where cannulas make the fluidic connection to the cover conical holes^19^ while control electronics multiplex each LED so that the entire array response is obtained. A micro-pump supplies reagents and analytes. The fluidic sequence is: start to arrow 1: washing solution; arrow 1 to 2: assay buffer; arrow 2 to 3: 1 nM streptavidin solution in assay buffer (20 min); arrow 3 to 4: assay buffer; arrow 4 to 5: 10 nM anti-mouse IgG antibody in assay buffer (20 min); arrow 5 to 6: assay buffer; and arrow 6 to end of run: washing solution.

**Figure 8:**
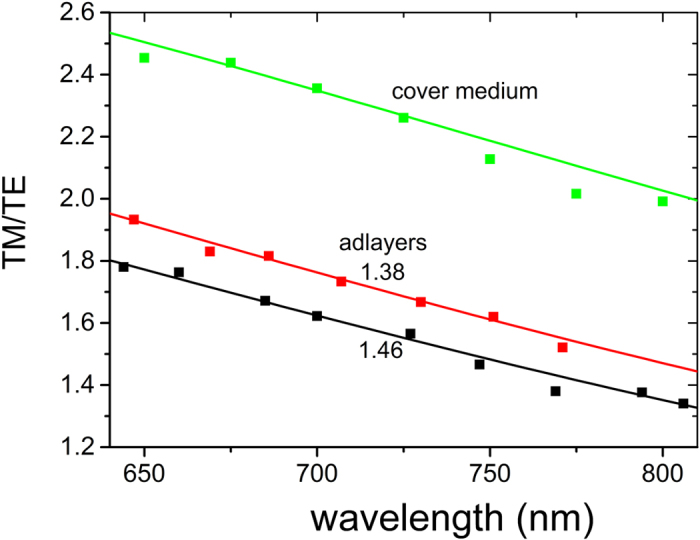
The ratio of the two polarization shifts, TM/TE, as a function of the wavelength for the anti-mouse and streptavidin binding reaction (red and black plots, respectively). The results include experimental measurements (scattered points) and theoretical calculations, δN_s2_/δN_s1_ ratios, (smooth curves) corresponding to adlayer refractive indices of 1.38 (anti-mouse, red) and 1.46 (streptavidin, black). The upper green curve is the TM/TE ratio due to cover medium changes from Fig. 3.

The results from this experiment are used for the purpose of the independent determination of the protein adlayer refractive index. As in Fig. 3, individual spectrometer channels are monitored following deconvolution of the TE and the TM polarization in the Fourier transform domain. The ratio of the TM over the TE phase shift is plotted as a function of the wavelength for the two proteins as shown in Fig. 8. For comparison the same ratio for the cover medium change in Fig. 3c, was also plotted. The experimental TM/TE ratio corresponding to anti-mouse IgG antibody is higher than the one corresponding to streptavidin. The cover medium TM/TE ratio is well above both and provides a measure on whether phase changes are due to an adlayer or a cover medium change. Theoretical TM/TE values, calculated through Eq. (5) as δN_s2_/δN_s1_ ratios, are also plotted. Here, δN_s2_ and δN_s1_ are the simulated sensing arm effective index changes due to an adlayer formation of a given thickness (5 nm). The adlayer effective indices were changed to obtain the best fits to experimental results. The fits correspond to 1.38 (anti-mouse IgG antibody) and 1.46 (streptavidin). The above analysis proves that the presented methodology, in addition to low detection limits, provides the analytical tools to optically characterize the adlayers resulting from multiple binding assays and at the same time distinguishes cover medium from adlayer induced spectral shifts.

## Discussion

A multiplexed silicon interferometric chip and an associated analytical method were presented. The chip monolithically integrates broad-band silicon LEDs coupled to monomodal silicon nitride waveguides forming Mach-Zehnder interferometers. The fundamental modes of either polarization are excited at the input and waveguided throughout the interferometers. As opposed to traditional resonators, the present device employs standard spectrometers as high resolution detectors which is possible due to the spectral shift enhancement inherent in Mach-Zehnder configurations. The broad-band nature of the waveguided light makes the simultaneous deconvolution of the TE and TM modes by signal processing of the modulated output light spectrum possible. The dual polarization analysis over a broad spectral band makes the refractive index calculation of the binding adlayers as well as the distinction of effective medium changes into cover medium or adlayer ones possible.

The limit of detection is by no means compromised by the broad light spectrum as it depends on the total number of the detected photons and the length of the exposed arm. It should also be noted that all experiments were performed in standard laboratory conditions without any particular temperature stabilization of the docking station. The pM range detection limits obtained with the proposed biosensor place it amongst the most sensitive label-free Mach-Zehnder interferometric sensors reported in literature. In particular, the developed sensor is 5 times less sensitive in terms of anti-rabbit IgG detection than a SU-8 MZI, which, however, has approximately 17 times longer sensing arm interaction length^27^. In terms of streptavidin, the proposed BB-MZI is approximately 550 and 60 times more sensitive compared to a polyimide-waveguide MZI^28^ and a two-lateral-mode spiral waveguide MZI^29^, respectively, both having much longer sensing arm lengths. The obtained sensitivities, as well as the projected ones for longer waveguides, allow for the detection of certain blood markers and contaminants in real samples. The detection limits presented here are considerably lower than the ones encountered in other label-free devices where spotted planar microarrays are read by cell phone cameras^21^,^22^. This is a result of the guided optics interferometry employed here. On the other hand, the interferometer length and the multiplexed read-out scheme place the limit on the number of readable analytes at few tens. This is not an issue for the aforementioned cell phone based devices where higher analyte numbers can be read simultaneously.

## Methods

### Materials

Bovine serum albumin (BSA), mouse gamma-globulins, goat anti-mouse IgG antibody and 3-aminopropyl-triethoxysilane (APTES) were purchased from Sigma Chemical Co. (St. Louis, MO, USA). Immunopure Streptavidin and EZ-Link Sulfo-NHS-LC-Biotin were from Pierce (Rockford, IL, USA). All other materials were purchased from Merck (Darmstadt, Germany). Biotinylated BSA (b-BSA) was prepared according to a previously published protocol^30^.

### Sensor chip and fluidic layout

The entire fabrication of the biosensor chips was realized in the clean room facility of the Institute of Nanoscience and Nanotechnology of NCSR “Demokritos” as it has been described in previous publications^18^,^19^,^26^. A schematic of the chip layout is depicted in Fig. 1b, showing the topological arrangement of the 10 BB-MZIs on the chip, the LED pads and the convergence of the 10 waveguides at the output signal collection area as well as the positioning of the fluidic cover (highlighted green). In the chip image provided in Fig. 1c, the arrangement of the 10 (600-micron long, 25-micron wide) sensing arm windows can be observed; the silicon nitride rib waveguides are not visible under a standard optical microscope. The specially-designed fluidic module consisted of a poly(methyl methacrylate) top layer with drilled inlet and outlet holes onto which a thick film adhesive photoresist was laminated and photolithographically patterned to create the flow channel. The fluidic covers are assembled either on single chips or on wafer scale through the adhesive photoresist defining the fluidic cell.

### Chip surface activation with APTES and deposition of biomolecules

Oxygen plasma cleaned/hydrophilized chips were immersed in a 0.5% (v/v) aqueous APTES solution for 2 min. Then, they were gently washed with distilled water, dried under a stream of nitrogen and cured for 20 min at 120˚C. Deposition of biomolecule solutions (mouse IgG or biotinylated-BSA 25 μg/mL in 50 mM phosphate buffer, pH 7.4) on BB-MZI chips was performed using the BioOdyssey Calligrapher Mini Arrayer (Bio-Rad Laboratories Inc., USA) equipped with a stainless steel pin with tip diameter of 375 microns purchased from Arrayit Corporation (Sunnyvale, CA, USA). Grids of 5 × 3 spots with center to center distance of 150 μm were deposited on each sensing window to ensure its complete coverage with the respective biomolecule solutions. After spotting, the substrates were incubated for at least 18 h under controlled temperature and humidity conditions (15˚C and 65% humidity). Afterwards, the spotted substrates were washed with 50 mM phosphate buffer, pH 7.4 (washing solution), and immersed in a 10 g/L BSA solution in 50 mM phosphate buffer, pH 7.4, (blocking solution), for 2 hours at room temperature, in order to cover the remaining free binding sites of the surface. The substrates were then washed with 50 mM phosphate buffer, pH 7.4, and distilled water, dried under a stream of nitrogen, and used for the assay.

### On-chip assays

Prior to the assay, the fluidic cover was attached on top of the biofunctionalized chips and the continuous delivery of the reagents solutions over the chip surfaces was achieved by means of a syringe pump (Cole Parmer 74900 series) providing the solutions at a constant flow of 20 μl/min. The encapsulated chip was placed on a handling frame and then to the docking station of the measuring instrument which provides both the electrical and the fluidic connections. For the dual analyte experiments, appropriate mixture of streptavidin and anti-mouse IgG antibody solutions at concentrations ranging from 2 pM to 2.5 nM for streptavidin and 25 pM to 66.7 nM for anti-mouse IgG antibody were prepared in 50 mM phosphate buffer, pH 7.4, containing 10 g/L BSA (assay buffer) and run for 20 min over chips equilibrated with assay buffer. Spectral acquisition was performed in a multiplexed way in a 10 s sampling cycle (1s per BB-MZI) through custom-made LabView-based software. The recorded spectra files were analyzed off-line and were subjected to Discrete Fourier Transform using a specially developed MatLab program.

## Additional Information

**How to cite this article**: Psarouli, A. *et al.* Monolithically integrated broad-band Mach-Zehnder interferometers for highly sensitive label-free detection of biomolecules through dual polarization optics. *Sci. Rep.* **5**, 17600; doi: 10.1038/srep17600 (2015).
